# Supplementary material for: How do local-level authorities engage in epidemic and pandemic preparedness activities and coordinate with higher levels of government? Survey results from 33 cities
Source: PLOS Glob Public Health. 2022 Oct 19;2(10):e0000650. doi: 10.1371/journal.pgph.0000650 (PMC10022361; doi:10.1371/journal.pgph.0000650)
Supplement: S1 Text — (PDF) [file pgph.0000650.s001.pdf]

S1 Text | Survey Questionnaire.

Section I: Background and demographic information

1. **What is your full name?**

2. **What is your gender?**
   A. Male B. Female
   C. Other D. Prefer not to answer

3. **What is your preferred email address?**

4. **What city or urban area do you represent? (City, Country)**

5. **What is the name of your department, office, or agency?**

6. **What is your job title?**

7. **How many years have you been in this role?**
   A. Less than 1 year B. 1-4 years
   C. 5-9 years D. 10 years or more

8. **If your role and title changed as a result of the response to the COVID-19 pandemic, what was the name of your department/office and job title before the pandemic?**

Section II: Epidemic/Pandemic Preparedness in Your City

1. **Which levels of government are responsible for the implementation of the following pandemic preparedness activities in your city? Please select all levels of government that are responsible for implementing an activity in your city.**

Preparedness Activity	Level of Government				
	National	Regional (State/Province)	Local (County/District)	Municipal (City)	Not Applicable
Developing pandemic preparedness and response policy					
Operationalizing pandemic preparedness and response policy					
Financing routine public health activities					
Developing mechanisms for coordination between levels of government					
Conducting routine infectious disease surveillance activities					
Conducting the initial investigation of infectious disease outbreaks					
Conducting emergency risk assessments and developing risk profiles					
Developing and maintaining pandemic preparedness and response plans					
Identifying and mapping resources required for the response to public health emergencies					
Conducting simulation or table-top exercises to test capacities					
Conducting after-action reviews following public health emergencies or events					

2. **Which level of government is accountable for the following pandemic preparedness activities in your city? Please select the level of government that oversees an activity in your city.**

Preparedness Activity	Level of Government				
	National	Regional (State/Province)	Local (County/District)	Municipal (City)	Not Applicable
Developing pandemic preparedness and response policy					
Operationalizing pandemic preparedness and response policy					
Financing routine public health activities					
Developing mechanisms for coordination between levels of government					
Conducting routine infectious disease surveillance activities					
Conducting the initial investigation of infectious disease outbreaks					
Conducting emergency risk assessments and developing risk profiles					
Developing and maintaining pandemic preparedness and response plans					
Identifying and mapping resources required for the response to public health emergencies					
Conducting simulation or table-top exercises to test capacities					
Conducting after-action reviews following public health emergencies or events					

3. **Which levels of government are consulted (i.e., provide information and with whom there is two-way communication) for the following pandemic preparedness activities in your city? Please select all levels of government that are consulted for an activity in your city.**

Preparedness Activity	Level of Government				
	National	Regional (State/Province)	Local (County/District)	Municipal (City)	Not Applicable
Developing pandemic preparedness and response policy					
Operationalizing pandemic preparedness and response policy					
Financing routine public health activities					
Developing mechanisms for coordination between levels of government					
Conducting routine infectious disease surveillance activities					
Conducting the initial investigation of infectious disease outbreaks					
Conducting emergency risk assessments and developing risk profiles					
Developing and maintaining pandemic preparedness and response plans					
Identifying and mapping resources required for the response to public health emergencies					
Conducting simulation or table-top exercises to test capacities					
Conducting after-action reviews following public health emergencies or events					

4. **Which levels of government are informed (i.e., informed of updates and with whom there is one-way communication) about the following pandemic preparedness activities in your city? Please select all levels of government that are informed about an activity in your city.**

Preparedness Activity	Level of Government				
	National	Regional (State/Province)	Local (County/District)	Municipal (City)	Not Applicable
Developing pandemic preparedness and response policy					
Operationalizing pandemic preparedness and response policy					
Financing routine public health activities					
Developing mechanisms for coordination between levels of government					
Conducting routine infectious disease surveillance activities					
Conducting the initial investigation of infectious disease outbreaks					
Conducting emergency risk assessments and developing risk profiles					
Developing and maintaining pandemic preparedness and response plans					
Identifying and mapping resources required for the response to public health emergencies					
Conducting simulation or table-top exercises to test capacities					
Conducting after-action reviews following public health emergencies or events					

5. **Had your city completed any of the following before the COVID-19 pandemic? Please select all that apply.**
   A. Infectious Disease Risk Assessment
   B. All-Hazards Risk Assessment
   C. Simulation or Table Top Exercises
   D. After-Action Reviews
   A. E. Other (Please specify)
   F. I don’t know/I am unsure

6. **If your city had completed a risk assessment, did it identify specific risks, vulnerabilities, or other factors (e.g., special populations) that might warrant special consideration during an emergency response? Please describe briefly.**

7. **Had your city developed a city-specific pandemic preparedness and response plan before the COVID-19 pandemic (e.g., influenza, Zika, all-hazards plan, etc.)?**
   A. Yes
   B. No
   C. I don’t know/I am unsure

8. **If possible, please provide a link to the plan(s).**

9. **Was your city involved or consulted during your country’s Joint External Evaluation (JEE) or in the development of a National Action Plan for Health Security (NAPHS)?**
   A. Yes, the Joint External Evaluation (JEE)
   B. Yes, the National Action Plan for Health Security (NAPHS)
   C. Yes, both the Joint External Evaluation (JEE) and the National Action Plan for Health Security (NAPHS)
   D. No, the city was not involved or consulted
   E. I don’t know/I am unsure
