# Supplementary material for: How do local-level authorities engage in epidemic and pandemic preparedness activities and coordinate with higher levels of government? Survey results from 33 cities
Source: PLOS Glob Public Health. 2022 Oct 19;2(10):e0000650. doi: 10.1371/journal.pgph.0000650 (PMC10022361; doi:10.1371/journal.pgph.0000650)
Supplement: S1 Data — (PDF) [file pgph.0000650.s002.pdf]

S1 Data | Survey Results.

Variable	Name	Values
city	City Name	NA
idra	Infectious Disease Risk Assessment	2 = Completed | 1 = Unsure | 0 = Not completed
ahra	All-Hazards Risk Assessment	2 = Completed | 1 = Unsure | 0 = Not completed
simex	Simulation Exercise	2 = Completed | 1 = Unsure | 0 = Not completed
aar	After-Action Review	2 = Completed | 1 = Unsure | 0 = Not completed
pprplan	PPR Plan	2 = Completed | 1 = Unsure | 0 = Not completed
jee	JEE	2 = Completed | 1 = Unsure | 0 = Not completed
naphs	NAPHS	2 = Completed | 1 = Unsure | 0 = Not completed

**city**	**idra**	**ahra**	**simex**	**aar**	**pprplan**	**jee**	**naphs**
Abidjan	1	1	1	1	2	2	2
Accra	0	0	2	0	0	2	2
Addis Ababa	2	0	0	0	2	0	2
Amman	2	2	0	0	2	0	2
Athens	1	1	1	1	1	1	1
Bandung	2	2	0	2	1	0	2
Bangkok	1	1	1	1	0	0	0
Barcelona	2	2	0	0	0	0	0
Bengaluru	1	1	1	1	0	0	0
Buenos Aires	2	2	2	2	2	0	0
Cali	2	1	2	1	2	1	1
Chicago	2	2	2	2	2	1	1
Colombo	2	2	0	2	2	0	2
Fortaleza	2	0	0	0	2	1	1
Guadalajara	2	0	0	0	2	1	1
Harare	2	0	2	0	2	1	1
Kampala	2	0	2	0	2	1	1
Kigali	0	0	0	0	0	0	2
Kumasi	2	1	1	1	2	2	2
Leon	1	1	1	1	2	1	1
Lima	2	0	2	0	1	1	1
London	2	2	2	2	2	1	1
Lusaka	0	0	0	2	2	1	1
Medellin	2	0	0	0	2	1	1
Melbourne	2	2	2	2	2	0	0
Montevideo	1	1	1	1	0	0	0
Ouagadougou	1	1	1	1	0	0	0
Quezon City	0	2	2	2	0	1	1
Rio de Janeiro	2	0	0	2	2	0	0
Santiago	2	0	0	2	2	1	1
Santo Domingo	0	0	0	0	0	0	0
Vancouver	0	2	0	0	1	0	0
Yangon	2	0	2	0	1	1	1
