# Supplementary material for: How do local-level authorities engage in epidemic and pandemic preparedness activities and coordinate with higher levels of government? Survey results from 33 cities
Source: PLOS Glob Public Health. 2022 Oct 19;2(10):e0000650. doi: 10.1371/journal.pgph.0000650 (PMC10022361; doi:10.1371/journal.pgph.0000650)
Supplement: S2 Data — (PDF) [file pgph.0000650.s003.pdf]

S2 Data | Urban Epidemic Preparedness RACI Matrices.

Legend		
**Governance Level**	National (N)	*National government*
	Subnational (S)	*State, provincial, district, or county governments*
	Local (L)	*City or municipal governments*
**RACI Roles**	Responsible (R)	*The level of government that implemented the work required to complete an activity*
	Accountable (A)	*The level of government that oversaw the correct and thorough completion of an activity*
	Consulted (C)	*Levels of government engaging in two-way communication to provide information necessary for the completion of an activity*
	Informed (I)	*Level of government that was updated regarding a given activity (i.e., one-way communication)*

**Abidjan, Côte d'Ivoire**	**Activity**	**N**	**S**	**L**
	Developing pandemic preparedness and response policy	RA	RC	RC
	Operationalizing pandemic preparedness and response policy	RC	RC	RA
	Financing routine public health activities	RC	RC	RA
	Developing mechanisms for coordination between levels of government	RA	RC	RC
	Conducting routine infectious disease surveillance activities	RA	RC	RC
	Conducting the initial investigation of infectious disease outbreaks	RA	RC	RC
	Conducting emergency risk assessments and developing risk profiles	RA	RC	RC
	Developing and maintaining pandemic preparedness and response plans	RA	RC	RC
	Identifying and mapping resources required for the response to public health emergencies	RA	RC	RC
	Conducting simulation or table-top exercises to test capacities	RA	I	I
	Conducting after-action reviews following public health emergencies or events	RA	I	I

**Accra, Ghana**	**Activity**	**N**	**S**	**L**
	Developing pandemic preparedness and response policy	RC	RA	RC
	Operationalizing pandemic preparedness and response policy	RA	RC	RC
	Financing routine public health activities	RA		
	Developing mechanisms for coordination between levels of government	RA	RI	R
	Conducting routine infectious disease surveillance activities		RA	
	Conducting the initial investigation of infectious disease outbreaks		RA	
	Conducting emergency risk assessments and developing risk profiles		RA	
	Developing and maintaining pandemic preparedness and response plans	R	RA	R
	Identifying and mapping resources required for the response to public health emergencies	R	RA	R
	Conducting simulation or table-top exercises to test capacities		RA	
	Conducting after-action reviews following public health emergencies or events		RA	

Addis Ababa, Ethiopia	Activity	N	S	L
	Developing pandemic preparedness and response policy	RA	RC	
	Operationalizing pandemic preparedness and response policy	RC	RA	RC
	Financing routine public health activities	RA	RC	RC
	Developing mechanisms for coordination between levels of government	RC	RA	RC
	Conducting routine infectious disease surveillance activities	RC	RA	C
	Conducting the initial investigation of infectious disease outbreaks	RA	RC	
	Conducting emergency risk assessments and developing risk profiles	RC	RA	
	Developing and maintaining pandemic preparedness and response plans	RA	RC	C
	Identifying and mapping resources required for the response to public health emergencies	RC	RA	
	Conducting simulation or table-top exercises to test capacities	RA	I	
	Conducting after-action reviews following public health emergencies or events	RA	RC	

Amman, Jordan	Activity	N	S	L
	Developing pandemic preparedness and response policy	RC		RA
	Operationalizing pandemic preparedness and response policy	RC		RA
	Financing routine public health activities	RA		RC
	Developing mechanisms for coordination between levels of government	RA		RC
	Conducting routine infectious disease surveillance activities	RA		RC
	Conducting the initial investigation of infectious disease outbreaks	RA		RC
	Conducting emergency risk assessments and developing risk profiles	RA	R	RC
	Developing and maintaining pandemic preparedness and response plans	RA	R	RC
	Identifying and mapping resources required for the response to public health emergencies	RA	R	RC
	Conducting simulation or table-top exercises to test capacities	RA	R	RC
	Conducting after-action reviews following public health emergencies or events	RA	RI	RC

Athens, Greece	Activity	N	S	L
	Developing pandemic preparedness and response policy	RC		A
	Operationalizing pandemic preparedness and response policy	RC	R	RA
	Financing routine public health activities	RC	R	RA
	Developing mechanisms for coordination between levels of government	RC	R	RA
	Conducting routine infectious disease surveillance activities	RA		
	Conducting the initial investigation of infectious disease outbreaks	RA		
	Conducting emergency risk assessments and developing risk profiles	RA		
	Developing and maintaining pandemic preparedness and response plans	RA	R	R
	Identifying and mapping resources required for the response to public health emergencies	RA	R	R
	Conducting simulation or table-top exercises to test capacities	I		
	Conducting after-action reviews following public health emergencies or events	RI		

Bandung, Indonesia	Activity	N	S	L
	Developing pandemic preparedness and response policy	RC	RC	RA
	Operationalizing pandemic preparedness and response policy	RC	RC	RA
	Financing routine public health activities	RA	RC	RC
	Developing mechanisms for coordination between levels of government	RC	RA	RC
	Conducting routine infectious disease surveillance activities	C	RC	RA
	Conducting the initial investigation of infectious disease outbreaks	C	RC	RA
	Conducting emergency risk assessments and developing risk profiles	RC	RC	RA
	Developing and maintaining pandemic preparedness and response plans	RC	RC	RA
	Identifying and mapping resources required for the response to public health emergencies	RC	RC	RA
	Conducting simulation or table-top exercises to test capacities	C	RC	RA
	Conducting after-action reviews following public health emergencies or events	RC	RC	RA

Bangkok, Thailand	Activity	N	S	L
	Developing pandemic preparedness and response policy	RA	RC	R
	Operationalizing pandemic preparedness and response policy	RA	RC	RC
	Financing routine public health activities	RA	RC	R
	Developing mechanisms for coordination between levels of government	RA	RC	
	Conducting routine infectious disease surveillance activities	RA	RC	R
	Conducting the initial investigation of infectious disease outbreaks	RA	RC	R
	Conducting emergency risk assessments and developing risk profiles	RA	RC	
	Developing and maintaining pandemic preparedness and response plans	RA	RC	R
	Identifying and mapping resources required for the response to public health emergencies	RA	RC	R
	Conducting simulation or table-top exercises to test capacities	RA	RI	
	Conducting after-action reviews following public health emergencies or events	RA	RC	R

Barcelona, Spain	Activity	N	S	L
	Developing pandemic preparedness and response policy	RA	RC	
	Operationalizing pandemic preparedness and response policy	RC	RA	
	Financing routine public health activities	R	RA	C
	Developing mechanisms for coordination between levels of government	RC	RA	R
	Conducting routine infectious disease surveillance activities		C	RA
	Conducting the initial investigation of infectious disease outbreaks		C	RA
	Conducting emergency risk assessments and developing risk profiles		C	RA
	Developing and maintaining pandemic preparedness and response plans	A	RC	
	Identifying and mapping resources required for the response to public health emergencies		A	RC
	Conducting simulation or table-top exercises to test capacities		A	RC
	Conducting after-action reviews following public health emergencies or events		A	RC

Bengaluru, India	Activity	N	S	L
	Developing pandemic preparedness and response policy	RC	RA	RC
	Operationalizing pandemic preparedness and response policy		RA	RC
	Financing routine public health activities		RA	RC
	Developing mechanisms for coordination between levels of government		RA	RC
	Conducting routine infectious disease surveillance activities		RA	RC
	Conducting the initial investigation of infectious disease outbreaks		RA	RC
	Conducting emergency risk assessments and developing risk profiles		RA	RC
	Developing and maintaining pandemic preparedness and response plans		RA	RC
	Identifying and mapping resources required for the response to public health emergencies		RA	RC
	Conducting simulation or table-top exercises to test capacities		RA	RC
	Conducting after-action reviews following public health emergencies or events	C	RA	RC

Buenos Aires, Argentina	Activity	N	S	L
	Developing pandemic preparedness and response policy	RC	RA	
	Operationalizing pandemic preparedness and response policy	I	RA	
	Financing routine public health activities	RC	RA	
	Developing mechanisms for coordination between levels of government	I	RA	
	Conducting routine infectious disease surveillance activities		RA	
	Conducting the initial investigation of infectious disease outbreaks		RA	
	Conducting emergency risk assessments and developing risk profiles		RA	
	Developing and maintaining pandemic preparedness and response plans	I	RA	
	Identifying and mapping resources required for the response to public health emergencies		RA	
	Conducting simulation or table-top exercises to test capacities		RA	
	Conducting after-action reviews following public health emergencies or events		RA	

Cali, Colombia	Activity	N	S	L
	Developing pandemic preparedness and response policy	RA	C	
	Operationalizing pandemic preparedness and response policy	RA	RC	RC
	Financing routine public health activities	RA	RC	RC
	Developing mechanisms for coordination between levels of government	RC	RA	R
	Conducting routine infectious disease surveillance activities	RC	RA	RC
	Conducting the initial investigation of infectious disease outbreaks	C	R	RA
	Conducting emergency risk assessments and developing risk profiles	C	RA	RC
	Developing and maintaining pandemic preparedness and response plans	A	RC	RC
	Identifying and mapping resources required for the response to public health emergencies	A	RC	RC
	Conducting simulation or table-top exercises to test capacities	RC	RA	RC
	Conducting after-action reviews following public health emergencies or events	A	RC	C

Chicago, USA	Activity	N	S	L
	Developing pandemic preparedness and response policy	RC	RC	RA
	Operationalizing pandemic preparedness and response policy	RC	RC	RA
	Financing routine public health activities	RA	RC	RC
	Developing mechanisms for coordination between levels of government	C	RC	RA
	Conducting routine infectious disease surveillance activities	C	RC	RA
	Conducting the initial investigation of infectious disease outbreaks	C	RC	RA
	Conducting emergency risk assessments and developing risk profiles		C	RA
	Developing and maintaining pandemic preparedness and response plans		C	RA
	Identifying and mapping resources required for the response to public health emergencies			RA
	Conducting simulation or table-top exercises to test capacities	RC	RC	RA
	Conducting after-action reviews following public health emergencies or events	RC	RC	RA

Colombo, Sri Lanka	Activity	N	S	L
	Developing pandemic preparedness and response policy	RA		
	Operationalizing pandemic preparedness and response policy	RA		
	Financing routine public health activities	RA		
	Developing mechanisms for coordination between levels of government	RA		R
	Conducting routine infectious disease surveillance activities	RC		RA
	Conducting the initial investigation of infectious disease outbreaks	RC		RA
	Conducting emergency risk assessments and developing risk profiles	RA		
	Developing and maintaining pandemic preparedness and response plans	RA		R
	Identifying and mapping resources required for the response to public health emergencies	RA		
	Conducting simulation or table-top exercises to test capacities	RA		
	Conducting after-action reviews following public health emergencies or events	RA		

Fortaleza, Brazil	Activity	N	S	L
	Developing pandemic preparedness and response policy	C	RA	RI
	Operationalizing pandemic preparedness and response policy	R	RA	RI
	Financing routine public health activities	A	RC	RI
	Developing mechanisms for coordination between levels of government	C	RA	RI
	Conducting routine infectious disease surveillance activities		RA	RI
	Conducting the initial investigation of infectious disease outbreaks	C	RA	RI
	Conducting emergency risk assessments and developing risk profiles		RA	RI
	Developing and maintaining pandemic preparedness and response plans	RC	RA	RI
	Identifying and mapping resources required for the response to public health emergencies	R	RA	RI
	Conducting simulation or table-top exercises to test capacities	RI	RA	I
	Conducting after-action reviews following public health emergencies or events	RC	RA	RI

Guadalajara, Mexico	Activity	N	S	L
	Developing pandemic preparedness and response policy	RA	RC	RC
	Operationalizing pandemic preparedness and response policy	RC	C	A
	Financing routine public health activities	RC	RC	RA
	Developing mechanisms for coordination between levels of government	RC	RA	RC
	Conducting routine infectious disease surveillance activities	RI	RC	A
	Conducting the initial investigation of infectious disease outbreaks	RC	RA	RC
	Conducting emergency risk assessments and developing risk profiles	RC	RA	RC
	Developing and maintaining pandemic preparedness and response plans	RA	RC	C
	Identifying and mapping resources required for the response to public health emergencies	RC	C	A
	Conducting simulation or table-top exercises to test capacities	RC	RC	RA
	Conducting after-action reviews following public health emergencies or events	RA	RC	RI

Harare, Zimbabwe	Activity	N	S	L
	Developing pandemic preparedness and response policy	RA	RC	RC
	Operationalizing pandemic preparedness and response policy		RC	RA
	Financing routine public health activities	RC	C	RA
	Developing mechanisms for coordination between levels of government		RA	C
	Conducting routine infectious disease surveillance activities		C	RA
	Conducting the initial investigation of infectious disease outbreaks	I	C	RA
	Conducting emergency risk assessments and developing risk profiles	C	RA	RC
	Developing and maintaining pandemic preparedness and response plans		RC	RA
	Identifying and mapping resources required for the response to public health emergencies	C	RC	RA
	Conducting simulation or table-top exercises to test capacities		RA	RC
	Conducting after-action reviews following public health emergencies or events	C	RC	RA

Kampala, Uganda	Activity	N	S	L
	Developing pandemic preparedness and response policy	RC	C	A
	Operationalizing pandemic preparedness and response policy	C	C	RA
	Financing routine public health activities	RC		RA
	Developing mechanisms for coordination between levels of government	RC		RA
	Conducting routine infectious disease surveillance activities	I		RA
	Conducting the initial investigation of infectious disease outbreaks	I		RA
	Conducting emergency risk assessments and developing risk profiles	I		RA
	Developing and maintaining pandemic preparedness and response plans	RC		RA
	Identifying and mapping resources required for the response to public health emergencies	RI		RA
	Conducting simulation or table-top exercises to test capacities	I		RA
	Conducting after-action reviews following public health emergencies or events	RI		RA

	Activity	N	S	L
**Kigali, Rwanda**	Developing pandemic preparedness and response policy	RA	RC	RC
	Operationalizing pandemic preparedness and response policy	C	RC	RA
	Financing routine public health activities	RC	I	RA
	Developing mechanisms for coordination between levels of government	A	RI	RI
	Conducting routine infectious disease surveillance activities	C	RI	RA
	Conducting the initial investigation of infectious disease outbreaks	A	RI	RC
	Conducting emergency risk assessments and developing risk profiles	RA	RI	RI
	Developing and maintaining pandemic preparedness and response plans	A	RI	RC
	Identifying and mapping resources required for the response to public health emergencies	RA	RI	RC
	Conducting simulation or table-top exercises to test capacities	RA	I	I
	Conducting after-action reviews following public health emergencies or events	A	RI	RC

	Activity	N	S	L
**Kumasi, Ghana**	Developing pandemic preparedness and response policy	RA	RC	RC
	Operationalizing pandemic preparedness and response policy	RA	RC	RC
	Financing routine public health activities	RA	C	RC
	Developing mechanisms for coordination between levels of government	RA	RC	RC
	Conducting routine infectious disease surveillance activities	RA	RC	RC
	Conducting the initial investigation of infectious disease outbreaks	C	C	RA
	Conducting emergency risk assessments and developing risk profiles	C	RC	RA
	Developing and maintaining pandemic preparedness and response plans	RC	RC	RA
	Identifying and mapping resources required for the response to public health emergencies	RA	C	RC
	Conducting simulation or table-top exercises to test capacities	A	C	RC
	Conducting after-action reviews following public health emergencies or events	RA	RC	RC

	Activity	N	S	L
**León, Mexico**	Developing pandemic preparedness and response policy	RC	RC	RA
	Operationalizing pandemic preparedness and response policy	C	RC	RA
	Financing routine public health activities	R	RC	RA
	Developing mechanisms for coordination between levels of government	RC	RC	RA
	Conducting routine infectious disease surveillance activities	R	RC	RA
	Conducting the initial investigation of infectious disease outbreaks		C	RA
	Conducting emergency risk assessments and developing risk profiles	R	RC	RA
	Developing and maintaining pandemic preparedness and response plans	R	RC	RA
	Identifying and mapping resources required for the response to public health emergencies	R	RC	RA
	Conducting simulation or table-top exercises to test capacities		C	RA
	Conducting after-action reviews following public health emergencies or events		C	RA

Lima, Peru	Activity	N	S	L
	Developing pandemic preparedness and response policy	RA	RC	RC
	Operationalizing pandemic preparedness and response policy	C	RC	RA
	Financing routine public health activities	RA	RC	RC
	Developing mechanisms for coordination between levels of government	RA	RC	RC
	Conducting routine infectious disease surveillance activities	RA	RC	C
	Conducting the initial investigation of infectious disease outbreaks	RA	RC	
	Conducting emergency risk assessments and developing risk profiles	RA	RC	
	Developing and maintaining pandemic preparedness and response plans	RA	RC	RC
	Identifying and mapping resources required for the response to public health emergencies	RA	RC	RC
	Conducting simulation or table-top exercises to test capacities	RA	RC	R
	Conducting after-action reviews following public health emergencies or events	RA	RC	RC

London, United Kingdom	Activity	N	S	L
	Developing pandemic preparedness and response policy	RC	RC	RA
	Operationalizing pandemic preparedness and response policy	C	RA	RC
	Financing routine public health activities	RC	RA	I
	Developing mechanisms for coordination between levels of government	RC	C	RA
	Conducting routine infectious disease surveillance activities	R	RA	I
	Conducting the initial investigation of infectious disease outbreaks	C	RA	I
	Conducting emergency risk assessments and developing risk profiles	C	RC	RA
	Developing and maintaining pandemic preparedness and response plans	RA	RC	RC
	Identifying and mapping resources required for the response to public health emergencies	RA	RC	RC
	Conducting simulation or table-top exercises to test capacities	C	RC	RA
	Conducting after-action reviews following public health emergencies or events	RA	C	RC

Lusaka, Zambia	Activity	N	S	L
	Developing pandemic preparedness and response policy	RA	C	C
	Operationalizing pandemic preparedness and response policy	RC	RA	RC
	Financing routine public health activities	RA	RC	I
	Developing mechanisms for coordination between levels of government	RA	RC	RC
	Conducting routine infectious disease surveillance activities	RC	RA	C
	Conducting the initial investigation of infectious disease outbreaks	RC	RA	C
	Conducting emergency risk assessments and developing risk profiles	RC	RA	C
	Developing and maintaining pandemic preparedness and response plans	RC	RA	C
	Identifying and mapping resources required for the response to public health emergencies	RC	A	C
	Conducting simulation or table-top exercises to test capacities	RA	C	I
	Conducting after-action reviews following public health emergencies or events	RA	RC	C

Medellín, Colombia	Activity	N	S	L
	Developing pandemic preparedness and response policy	RA	C	C
	Operationalizing pandemic preparedness and response policy	C	RC	RA
	Financing routine public health activities	RC	RC	RA
	Developing mechanisms for coordination between levels of government	RC	RA	RC
	Conducting routine infectious disease surveillance activities	I	RC	RA
	Conducting the initial investigation of infectious disease outbreaks	I	RC	RA
	Conducting emergency risk assessments and developing risk profiles	I	RC	RA
	Developing and maintaining pandemic preparedness and response plans	RA	RC	RC
	Identifying and mapping resources required for the response to public health emergencies	RC	RC	RA
	Conducting simulation or table-top exercises to test capacities	C	RC	RA
	Conducting after-action reviews following public health emergencies or events	RC	RC	RA

Melbourne, Australia	Activity	N	S	L
	Developing pandemic preparedness and response policy	R	RC	RA
	Operationalizing pandemic preparedness and response policy		RC	RA
	Financing routine public health activities		RC	RA
	Developing mechanisms for coordination between levels of government	R	RA	RC
	Conducting routine infectious disease surveillance activities	R	RA	RC
	Conducting the initial investigation of infectious disease outbreaks		RA	C
	Conducting emergency risk assessments and developing risk profiles		RA	C
	Developing and maintaining pandemic preparedness and response plans		RC	RA
	Identifying and mapping resources required for the response to public health emergencies		RC	RA
	Conducting simulation or table-top exercises to test capacities		RC	RA
	Conducting after-action reviews following public health emergencies or events		RC	RA

Montevideo, Uruguay	Activity	N	S	L
	Developing pandemic preparedness and response policy	RA	RC	
	Operationalizing pandemic preparedness and response policy	RA	RC	
	Financing routine public health activities	RA	RC	
	Developing mechanisms for coordination between levels of government	RA	RC	
	Conducting routine infectious disease surveillance activities	RA	C	
	Conducting the initial investigation of infectious disease outbreaks	RA	C	
	Conducting emergency risk assessments and developing risk profiles	RA	C	
	Developing and maintaining pandemic preparedness and response plans	RA	C	
	Identifying and mapping resources required for the response to public health emergencies	RA	RC	
	Conducting simulation or table-top exercises to test capacities	RA	C	
	Conducting after-action reviews following public health emergencies or events	RA	C	

Ouagadougou, Burkina Faso

Activity	N	S	L
Developing pandemic preparedness and response policy	RA	C	RC
Operationalizing pandemic preparedness and response policy	A	RC	RC
Financing routine public health activities	RC		RA
Developing mechanisms for coordination between levels of government	RA	C	C
Conducting routine infectious disease surveillance activities	RA	R	
Conducting the initial investigation of infectious disease outbreaks	RA		I
Conducting emergency risk assessments and developing risk profiles	RA		RI
Developing and maintaining pandemic preparedness and response plans	RA	I	RC
Identifying and mapping resources required for the response to public health emergencies	RA	I	RC
Conducting simulation or table-top exercises to test capacities	RA	I	
Conducting after-action reviews following public health emergencies or events	RA	I	

Quezon City, Philippines

Activity	N	S	L
Developing pandemic preparedness and response policy	RC	RC	RA
Operationalizing pandemic preparedness and response policy	RC	RC	RA
Financing routine public health activities	R	RC	RA
Developing mechanisms for coordination between levels of government	RC	RC	RA
Conducting routine infectious disease surveillance activities	I	RI	RA
Conducting the initial investigation of infectious disease outbreaks	I	RI	RA
Conducting emergency risk assessments and developing risk profiles	R	RI	RA
Developing and maintaining pandemic preparedness and response plans	R	RC	RA
Identifying and mapping resources required for the response to public health emergencies	RC	RC	RA
Conducting simulation or table-top exercises to test capacities	RC	RC	RA
Conducting after-action reviews following public health emergencies or events	RC	RC	RA

Rio de Janeiro, Brazil

Activity	N	S	L
Developing pandemic preparedness and response policy	RC	RC	RA
Operationalizing pandemic preparedness and response policy	RC	RC	RA
Financing routine public health activities	RA	RC	R
Developing mechanisms for coordination between levels of government	RA	C	C
Conducting routine infectious disease surveillance activities	RC	RC	RA
Conducting the initial investigation of infectious disease outbreaks	RC	RC	RA
Conducting emergency risk assessments and developing risk profiles	RA	RC	RC
Developing and maintaining pandemic preparedness and response plans	RA	RC	RC
Identifying and mapping resources required for the response to public health emergencies	RC	RA	RC
Conducting simulation or table-top exercises to test capacities	RA	RC	RC
Conducting after-action reviews following public health emergencies or events	RA	RC	RC

Santiago, Chile

Activity	N	S	L
Developing pandemic preparedness and response policy	RA	C	
Operationalizing pandemic preparedness and response policy	C	RA	R
Financing routine public health activities	RA	RI	R
Developing mechanisms for coordination between levels of government	C	RA	
Conducting routine infectious disease surveillance activities	C	RA	RC
Conducting the initial investigation of infectious disease outbreaks	C	RA	R
Conducting emergency risk assessments and developing risk profiles	RC	RA	
Developing and maintaining pandemic preparedness and response plans	RC	RA	RC
Identifying and mapping resources required for the response to public health emergencies	RC	RA	RC
Conducting simulation or table-top exercises to test capacities	I	RA	RC
Conducting after-action reviews following public health emergencies or events	RA	RC	

Santo Domingo, Dominican Republic

Activity	N	S	L
Developing pandemic preparedness and response policy	RC	C	A
Operationalizing pandemic preparedness and response policy	RC	RC	RA
Financing routine public health activities	RC	I	RA
Developing mechanisms for coordination between levels of government	A	RC	R
Conducting routine infectious disease surveillance activities	RI	RA	R
Conducting the initial investigation of infectious disease outbreaks	RA	RI	
Conducting emergency risk assessments and developing risk profiles	RA	RC	
Developing and maintaining pandemic preparedness and response plans	RA	RC	R
Identifying and mapping resources required for the response to public health emergencies	RC	RA	
Conducting simulation or table-top exercises to test capacities	RA	RC	R
Conducting after-action reviews following public health emergencies or events	RA	RC	R

Vancouver, Canada

Activity	N	S	L
Developing pandemic preparedness and response policy	R	RA	RC
Operationalizing pandemic preparedness and response policy	R	RA	RC
Financing routine public health activities		RA	
Developing mechanisms for coordination between levels of government	R	RA	RC
Conducting routine infectious disease surveillance activities	I	RA	
Conducting the initial investigation of infectious disease outbreaks	I	RA	
Conducting emergency risk assessments and developing risk profiles		RA	RC
Developing and maintaining pandemic preparedness and response plans	R	RA	I
Identifying and mapping resources required for the response to public health emergencies	RC	RA	I
Conducting simulation or table-top exercises to test capacities	R	RA	R
Conducting after-action reviews following public health emergencies or events		RA	

	Activity	N	S	L
Yangon, Myanmar	Developing pandemic preparedness and response policy	RA		
	Operationalizing pandemic preparedness and response policy	RC	C	
	Financing routine public health activities			
	Developing mechanisms for coordination between levels of government	A		
	Conducting routine infectious disease surveillance activities			
	Conducting the initial investigation of infectious disease outbreaks	A		
	Conducting emergency risk assessments and developing risk profiles	A		
	Developing and maintaining pandemic preparedness and response plans			
	Identifying and mapping resources required for the response to public health emergencies			
	Conducting simulation or table-top exercises to test capacities			
	Conducting after-action reviews following public health emergencies or events	A		
